# Climbing the ladder: toward a robust and diverse physician-scientist workforce

**DOI:** 10.1172/JCI162080

**Published:** 2022-12-15

**Authors:** Jose A. Rodrigues

**Affiliations:** DO-PhD Trainee, Michigan State University College of Osteopathic Medicine, East Lansing, Michigan, USA. President, American Physician Scientists Association.

I distinctly remember attending my first American Association of Physicians (AAP), American Society for Clinician Investigation (ASCI), and American Physician Scientists Association (APSA) Joint Meeting in 2016. One of the first things I had done was bump into someone I later found out was a Nobel Prize laureate. After a flustered recovery, I had a short conversation and left the interaction feeling encouraged and excited to explore the science. The AAP/ASCI/APSA Joint Meeting itself was inspiring — I met several colleagues, including people I now consider friends, who have already impacted the trajectory of my life and career.

Reflecting upon my experience, I thought about what an incredible opportunity it was to elevate and inspire others. I believed APSA could continue providing opportunities to level the playing field and incorporate equity, inclusion, and advancement into our mentorship and networking. I thought, “How do we get more folks like me to crash into Nobel laureates?”

Over the course of my years of service with the American Physician Scientists Association, I have sought opportunities to bridge gaps between my peers and those aspiring to become physician-scientists. As I continued my training, I came across the Sherry R. Arnstein Scholarship from the American Association of Colleges of Osteopathic Medicine (AACOM). I found Sherry Arnstein’s biography to be compelling. Ms. Arnstein led AACOM as executive director for ten years. Prior to this appointment, she worked within the Kennedy administration and led efforts to desegregate all hospitals in the United States. However, she is most famous for her article entitled “A ladder of citizen participation,” published in 1969 (1). In her writing, she describes the rungs in which community members and planners interact using the analogy of rungs of a ladder. In this analogy, she describes citizen participation, delegation, and citizen control at the top of the ladder. I was compelled to think on how APSA exerts citizen participation, delegation, and control to create a robust and diverse physician-scientist workforce (Figure 1).

One instance was APSA’s partnership with the Burroughs Wellcome Fund to host the 2019 Physician-Scientist Diversity Summit, which engaged members of the physician-scientist community using design thinking. As a result of the summit, APSA was awarded a Power A Silver Award from the American Society for Association Executives (ASAE) in the category of diversity, equity, and inclusion. Putting participants first was the framework of the partnership, which we highlighted and used throughout all conference design (2).

This work eventually led to the formation of our Justice, Equity, Diversity, and Inclusion Committee (JEDI). JEDI has become a core component of APSA as the organization grows and develops within its mission to support the development of a robust and diverse physician-scientist workforce. APSA has been working to implement the solutions developed at the summit and within the JEDI community, focusing on initiatives such as financial literacy for physician-scientist trainees, supporting community college trainees, and a critical applicant interactive series.

Our virtual content committee has been leading efforts to support undergraduate trainees through the difficult process of applying to dual-degree programs through these interactive series. Since its development, the virtual content committee has hosted 21 sessions with an average of 130 participants. Additionally, members of this committee have worked with our partners – firstly with the Association of American Medical Colleges (AAMC), and more recently, the Society for Pediatric Research, the Association of Medical School Pediatric Department Chairs, and the National Institutes of Health. This committee works to create content meant to support applicants, current trainees, and early career physician-scientists within their early careers, including informational sessions, networking, and navigating the physician-scientist field. Additionally, we have hosted six virtual regional meetings in partnership with diverse institutions, from Pennsylvania to Oregon and from Texas to Nebraska and Tennessee.

Another example of citizen control in practice was the initiation development and sustained effort behind the Virtual Summer Research Program (VSRP). VSRP began in 2020 to address research opportunities that were canceled as a result of the coronavirus disease 2019 (COVID-19) pandemic. In the initial year, APSA was able to match 156 mentees from across the country with research opportunities. It was a herculean effort for which APSA was awarded yet another ASAE Power A Silver Award in the category of diversity and inclusion.

With a sustained partnership and support from the Burroughs Wellcome Fund, APSA has collaborated with regional institutions to coordinate events focused on recruitment and retention of individuals who have historically been excluded and underrepresented in medicine. These events took place in California, led by trainees at Stanford University and the University of California San Francisco, and there was a second event in Michigan led by trainees at Michigan State University. This culminated in a full week of events from May 24th through 28th, 2021, termed APSA Spotlight Week, which was also a partnership between multiple institutions and organizations, including sessions hosted in collaboration with the American Association of Black Physician Scientists (AABPS) and the Medical Student Pride Alliance (MSPA).

Persistence in the face of oppressive challenges has been a theme in our community over the past few years, and addressing challenges during the pandemic has been a major focus of the organization. APSA has adapted quickly, supporting trainees while they were persistent through the challenges of the pandemic, including with studies looking at the impact of the COVID-19 pandemic on current physician-scientist training (3).

APSA is further representing physician-scientist trainees across the country and internationally as a member of the International Consortium of Clinical Scientist Training Organizations (ICCTSO) and as a thought leader within academic medicine through participation with organizations such as the American Medical Association. We are also represented as a member of the Group on Research Education and Training and a member of the Training Opportunities for Physician-Scientists (TOPS) within the AAMC and the Physician-Scientist Development Committee within ASCI.

As the first osteopathic-trained medical student to lead the American Physician Scientists Association, it is a privilege to highlight the work of my fellow physician-scientists in the light and clarity of a framework focused on citizen engagement, control, and delegated power.

Over the course of my service with APSA, I’ve become a member of a community of practice and have had the humbling opportunity to support my peers in service leadership. It has been an honor and has given me great vocational pride to lead in service with this association. It has endowed in me skills via challenges that I would not have encountered elsewhere in my scientific training. I thoroughly encourage all to engage with APSA to participate and take control of the physician-scientist community (4). Ultimately, I know APSA will remain an organization critical to supporting the development of a diverse and robust physician-scientist workforce.

## Figures and Tables

**Figure 1:**
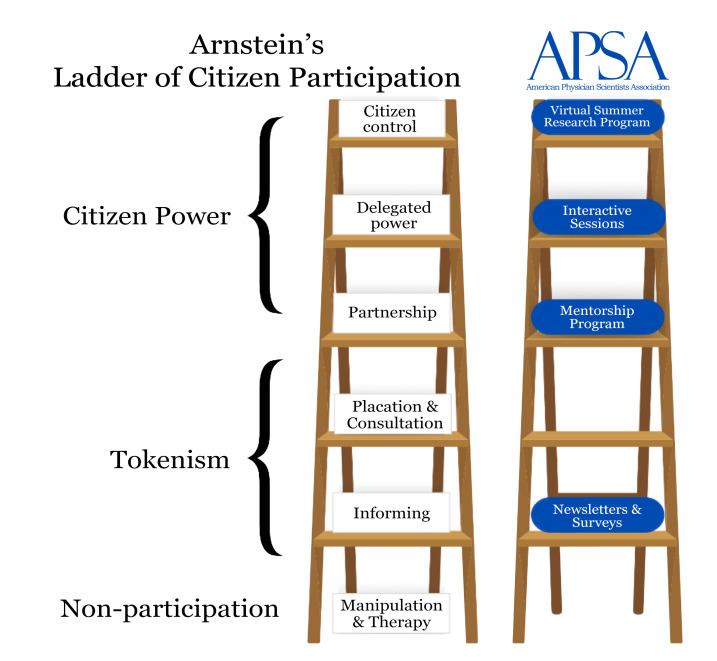
Adapted ladder of citizen participation. Arnstein’s ladder of citizen participation with subjective placement of highlighted programming from the American Physician Scientists Association based on the member engagement and inception of the programming. Adapted with permission from Organizing Engagement (1).

## Version 1 12/15/2022

Electronic publication
